# A new genus of the tribe Issini (Hemiptera, Fulgoromorpha, Issidae) from China

**DOI:** 10.3897/zookeys.228.3465

**Published:** 2012-10-12

**Authors:** Yanli Che, Yalin Zhang, Yinglun Wang

**Affiliations:** 1 Key Laboratory of Plant Protection Resources and Pest Management, Ministry of Education, Entomological Museum, Northwest A & F University, Yangling, Shaanxi 712100, China; 2 College of Plant Protection, Southwest University, Beibei, Chongqing 400716, China

**Keywords:** Taxonomy, Fulgoromorpha, *Macrodaruma*, new species

## Abstract

*Macrodarumoides petalinus*, a new genus and species of Issidae (Hemiptera) are described based on specimens from Yunnan and Guangxi, China.

## Introduction

Issidae are small insects (about 5mm to 20 mm) generally with a stocky body and usually brownish in color, few bright; and some have pronounced maculae. Issid planthoppers and ladybirds exhibit considerable similarity in general especially in the stocky body form. Issids are all plant feeders, with both nymphs and adults intaking phloem fluid from young branches and sometimes roots. Feeding of issids can result in the yellowing of plant foliage. At high population, issids can seriously affect plant growth (personal observation). Although they often have developed wings, some of them like to crawl and jump in shrubs, rather than to fly (personal observation). The family includes 973 species in 155 genera (Gnezdilov 2010).

Gnezdilov (2009) considered that the presence of the trilobed hind wing was an insufficient character for defining the tribe Thioniini, and therefore treated Thioniini Melichar, 1906 as a junior synonym of Issini Spinola, 1839. According to Gnezdilov (2003a, 2009), the subfamily Issinae consists of four tribes: Hemisphaeriini Melichar; Parahiraciini Cheng & Yang; Issini Spinola and Colpopterini Gnezdilov. In the present paper, a new genus and species, *Macrodarumoides petalinus*gen. et sp. n., from China is described and illustrated. Based on the hemispherical body, the claval suture on tegmen, the trilobed hind wings and the not-dilated legs,*Macrodarumoides* gen. n. is placed in the tribe Issini, which has the only species *Macrodarumoides petalinus*sp. n. from China.

## Materials and methods

The terminology of the head, body and male genitalia follows Chan and Yang (1994), and the terminology of the female genitalia follows Gnezdilov (2003b). The genital segments of the examined specimens were macerated in 10% KOH and observed in glycerin jelly using a Leica MZ125 stereomicroscope. Photographs of the specimens were made using a Nikon SMZ1500 stereomicroscope with a Q-image CCD. Images were produced using the software Synoptics Automontage. All the specimens studied are deposited in the Entomological Museum of Northwest Agriculture and Forestry University of (NWAFU) or the College of Life Sciences, Nankai University (NKU), as indicated.

### Macrodarumoides gen. n.

urn:lsid:zoobank.org:act:B8E3CFCA-C711-46F3-BBD1-E636BF9070A8

http://species-id.net/wiki/Macrodarumoides

#### Type species.

*Macrodarumoides petalinus* sp. n.

#### Description.

**Figures 1–6.**
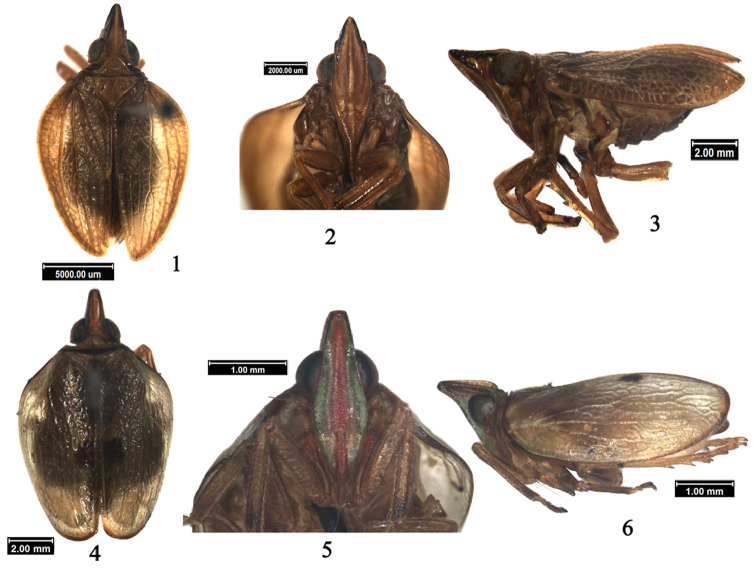
*Macrodarumoides petalinus* sp. n., male **1** Holotype, dorsal view **2** Holotype, head, ventral view **3** Holotype, lateral view; *Macrodaruma pertinax* Fennah, male: **4** Dorsal view **5** Head, ventral view **6** Lateral view.

**Figures 7–16.**
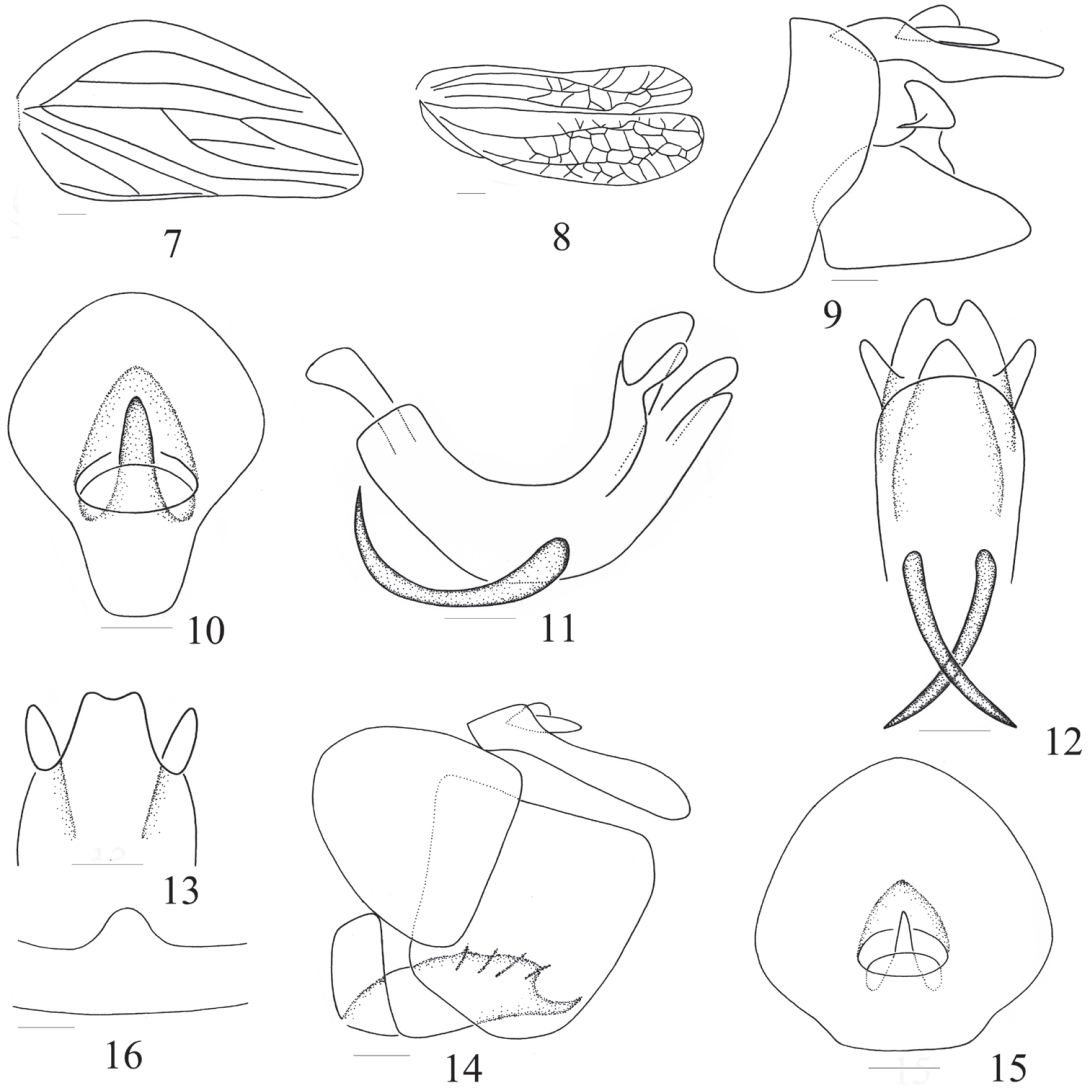
*Macrodarumoides petalinus* sp. n. **7** Tegmen **8** Wing **9** Male genitalia, left side **10** Male anal segment, dorsal view **11** Aedeagus, lateral view **12** Apex of aedeagus, ventral view **13** Apex of dorsal lobe, dorsal view **14** Female genitalia, left side **15** Female anal segment, dorsal view **16** Pregenital sternite, ventral view. Scale bars = 1.0 mm (Figs 7–8), 0.5 mm (Figs 9–16)

Head (including eyes) distinctly narrower than pronotum (Fig. 1). Vertex long and horizontal, approximately triangular, disc depressed, with 2 depressions near hind margin; anteriorly strongly angularly convex and posteriorly slightly angularly concave, lateral margins carinate; width at apex distinctly shorter than length in midline (Fig. 1). In dorsal view, vertex and frons extending far beyond eyes (Fig. 1). Ocelli present. Frons long and nearly triangular, disc obviously elevated, median carina present (Fig. 2); in lateral view frons curved towards apex (Fig. 3). Clypeus elevated with central carina, situated the same plane as frons (Fig. 2). Rostrum long, reaching to hind-trochanter. Pronotum short laterally, anterior margin convex and arched, posterior margin horizontal to slightly convex; disc elevated with pits (Fig. 1). Mesonotum nearly triangular, with 2 pits along lateral margins; disc slightly elevated, with or without carina (Fig. 1). Tegmen (Figs 1, 3, 7) leathery and approximately elliptical, claval suture present; longitudinal veins prominent, between them with a number of obscure veinlets, rendering the whole surface faintly reticulate. Wing (Fig. 8) large, veins distinct and netlike, longer than half of tegmen, apically forming 3 lobes. Legs (Fig. 3) relatively long, not dilated; lateral margin of hind tibia with 2 teeth. Spinal formula of hind leg (5-6)- 11-2, indicating number of spines at apex of hind tibia and tarsomeres I and II.

Male genitalia symmetrical; anal segment (Fig. 10) in dorsal view longer than greatest width; pygofer (Fig. 9) without spines; aedeagus (Figs 11-13) tubular, symmetrical and shallowly U-shaped, divided distally into a dorsal and ventral lobe, the latter sometimes split, a pair of elongate lateral lobes and a pair of spiniform processes lying ventrolaterally and directed either cephalad or caudad. Genital style (Fig. 9) subtrianglar, apical margin curved and arched, basal margin convex near apex and dorsal margin produced into a single process.

Female genitalia withanal segment (Fig. 15) in dorsal view elliptical, length nearly equal to the widest part. Apex of endogonocoxal process without lobe and anterior connective lamina of gonapophyse VIII with 3 teeth in lateral group. First valvula (Fig. 14) with teeth, ninth tergum and third valvula subquadrate. Pregenital sternite (Fig. 16) with apical margin convex at mid.

#### Diagnosis. 

This genus is similar to *Eusarima* Yang, 1994 according to body shape and trilobed wings, but can be differentiated by the following characteristics: 1) vertex long, approximately triangular, the latter with vertex short and subquadrangular; 2) wings narrow, netlike and anal lobe rudimentary, the latter, wings broad, longitudinal veins distinct with little cross veins, and anal lobe developed.

This genus resembles *Macrodaruma* Fennah, 1978 (Figs 4–6), but can be distinguished from the latter by: 1) lateral margins of vertex and anterior margin of pronotum not carinated, the latter, distinctly and foliately carinated; 2) tegmen with claval suture present, the latter, claval suture absent; 3) wing trilobed, the latter, not bilobed.

#### Etymology.

This generic name “-oides” from Greece suffix refers to the resemblance of this genus to *Macrodaruma* Fennah. The gender is masculine.

#### Distribution.

China (Guangxi, Yunnan).

### Macrodarumoides petalinus sp. n.

urn:lsid:zoobank.org:act:6D96BEEB-3639-41BC-88EA-43A0FD893343

http://species-id.net/wiki/Macrodarumoides_petalinus

Figs 1–3 7–16

#### Description.

Length, male (including tegmen): 8.1mm, length of tegmen: 6.0mm; female (including tegmen): 8.3mm, length of tegmen: 6.1mm.

Body brown (Fig. 1). Vertex with disc brown and lateral margins black. Eyes black brown. Frons brown with median carina paler and lateral margin black at apical half. Clypeus brown, rostrum pale brown. Pronotum, mesonotum, tegmen and legs brown; wing dark brown. Abdomen ventrally and dorsally brown, disc dark brown.

Vertex long and horizontal, approximately triangular, disc depressed, with 2 despressions near hind margin (Fig. 1); vertex 1.8× wider at apex than length in midline. Frons with disc distinctly elevated, with median carina (Fig. 2); oblique between median carina and lateral margin, frons curved towards apex in lateral view (Fig. 3), 1.1× wider at widest part than at base, 2.8× longer in midline than at widest part. Frontoclypeal suture nearly straight (Fig. 2). Thorax (Fig. 1): disc of pronotum with 2 pits; mesonotum short and broad, greatest width 1.7× medial length. Tegmen 2.5× longer than widest part; Sc long, reaching beyond midlength; Sc and R forked near apex, M 4-branched, Cu not forked, claval suture only reaching to middle of sutural margin (Figs 1, 3, 7). Wing 0.7× length of tegmen, veins distinctly reticulate (Fig. 8). Spinal formula of hind leg 11-(5-6)-2.

**Male genitalia.** Anal segment in dorsal view (Fig. 10) distinctly longer than greatest width apically, apical margin strongly convex, lateral margin strongly divergent from base to apex, anal tube situated near middle; in lateral view ventral margin convex at midlength, nearly truncate in distal half (Fig. 9). Pygofer in lateral view with hind margin evenly convex near middle and slightly convex at base (Fig. 9). Phallus in profile shallowly curved with 2 long spiniform processes directed cephalad at midlength. Aedeagus in profile with apex bifurcated (Fig. 11); dorsal lobe (Figs 11, 13) in dorsal view tri-lobed near apex, with apical margin slightly concave at mid, lateral margins curved downward and encasing lateral and ventral lobe; lateral lobes in lateral view divided and tapered into fingers (Fig. 11); ventral lobe with apical margin convex and arched at mid, and lateroapical angle rounded in ventral view (Fig. 12). Genital styles in lateral view nearly triangular, apical margin curved and arched, dorsal margin produced into one large process near apex; base of process acuminate and apex obtusely rounded in caudal view (Fig. 9).

**Female genitalia.** Anal segment in dorsal view (Fig. 15) slightly longer than greatest width with lateral margins convex, apical margin convex and arched, anal tube situated at basal half; in lateral view ventral margin concave at base, nearly truncate in distal half (Fig. 14). Ovipositor with anterior connective lamina of gonapophyse VIII curved dorsally, with 5 nearly parallel spines; tooth near lateral margin larger. Gonoplac stout, strongly convex and subquadrate, with apical margin polished (Fig. 14). Pregenital sternite with apical margin distinctly convex at midlength (Fig. 16).

#### Material examined.

*Holotype*, male, China: Yunnan, Mt. Baoshan, 1900m, 20 November 1999, coll. Qin Daozheng (NWAFU). *Paratypes*, one male, one female, China: Guangxi, Leye, Tonglelinchang, 15 September 1980, coll. Lu Junsheng (NWAFU); one female China: Guangxi, Leye, Yachangyanpeng, 24 September 1980, coll. Lu Junsheng (NWAFU); one female, same data as holotype (NWAFU); two males, one female, China: Yunnan, Mt. Baoshan, 22 August 1979, coll. Cui Jianxin (NKU).

#### Remarks.

This species can be differentiated from *Eusarima contorta* Yang, 1994 by the following characteristics: 1) vertex long, approximately triangular, the latter with vertex short and subquadrangular; 2) frons only with median carina, the latter with median and lateral carinae; 3) wings narrow, netlike and anal lobe rudimentary, the latter, wings broad, longitudinal veins distinct with little cross veins, and anal lobe developed.

This species resembles *Macrodaruma pertinax* Fennah, 1978 (Figs 4-6) in shape, but differs from the latter in the following characteristics: 1) lateral margins of vertex and lateral margins of pronotum not elevated, the latter with lateral margins of vertex and lateral margins of pronotum elevated foliately, 2) claval suture present, the latter without claval suture, 3) wing with 3 lobes, the latter with wing not split.

#### Etymology.

The specific name is derived from the Latin word “petalinus”, referring to the dorsal lobe of aedeagus being concave with the lateral margin distinctly reflected as a petal.

## Supplementary Material

XML Treatment for Macrodarumoides

XML Treatment for Macrodarumoides petalinus

## References

[B1] Chan ML Yang CT (1994) Issidae of Taiwan (Homoptera: Fulgoroidea). Chen Chung Book, Taichung, 188 pp.

[B2] Fennah RG (1978) Fulgoroidea (Homoptera) from Vietnam. Annales Zoologici 34 (9): 207-279.

[B3] Gnezdilov VM (2003a) A new tribe of the family Issidae with comments on the family as a whole (Homoptera: Cicadina). Zoosystematica Rossica 11: 305-309.

[B4] Gnezdilov VM (2003b) Review of the family Issidae (Homptera: Cicadina) of the European fauna with notes on the structure of ovipositor in the planthoppers. Chteniya Pamyati Nikolaya Aleksandrovicha Kholodkovskogo 56 (1): 1-145.

[B5] Gnezdilov VM (2009) Revisionary notes on some tropical Issidae and Nogodinidae (Hemiptera: Fulgoroidea). Acta Entomological Musei Nationalis Pragae 49: 75-92.

[B6] Gnezdilov VM (2010) Composition and distribution of the family Issidae (Hemiptera, Fulgoroidea). 13th International Auchenorrhyncha congress, Vaison-la-Romaine, France 28 June – 2 July 2010. Abstracts: talks and posters: 87–88.

